# Purification and Characterization of the Isoprene Monooxygenase from *Rhodococcus* sp. Strain AD45

**DOI:** 10.1128/aem.00029-22

**Published:** 2022-03-14

**Authors:** Leanne P. Sims, Colin W. J. Lockwood, Andrew T. Crombie, Justin M. Bradley, Nick E. Le Brun, J. Colin Murrell

**Affiliations:** a School of Environmental Sciences, University of East Anglia, Norwich, United Kingdom; b School of Chemistry, University of East Anglia, Norwich, United Kingdom; University of Illinois at Urbana-Champaign

**Keywords:** *Actinobacteria*, soluble diiron monooxygenase, isoprene metabolism, volatile organic compounds

## Abstract

Isoprene (2-methyl-1,3-butadiene) is a climate-active gas released to the atmosphere in large quantities, comparable to methane in magnitude. Several bacteria have been isolated which can grow on isoprene as a sole carbon and energy source, but very little information is available about the degradation of isoprene by these bacteria at the biochemical level. Isoprene utilization is dependent on a multistep pathway, with the first step being the oxidation of isoprene to epoxy-isoprene. This is catalyzed by a four-component soluble diiron monooxygenase, isoprene monooxygenase (IsoMO). IsoMO is a six-protein complex comprising an oxygenase (IsoABE), containing the di-iron active site, a Rieske-type ferredoxin (IsoC), a NADH reductase (IsoF), and a coupling/effector protein (IsoD), homologous to the soluble methane monooxygenase and alkene/aromatic monooxygenases. Here, we describe the purification of the IsoMO components from *Rhodococcus* sp. AD45 and reconstitution of isoprene-oxidation activity *in vitro*. Some IsoMO components were expressed and purified from the homologous host *Rhodococcus* sp. AD45-ID, a *Rhodococcus* sp. AD45 strain lacking the megaplasmid which contains the isoprene metabolic gene cluster. Others were expressed in Escherichia coli and purified as fusion proteins. We describe the characterization of these purified components and demonstrate their activity when combined with *Rhodococcus* sp. AD45 cell lysate. Demonstration of IsoMO activity *in vitro* provides a platform for further biochemical and biophysical characterization of this novel soluble diiron center monooxygenase, facilitating new insights into the enzymatic basis for the bacterial degradation of isoprene.

**IMPORTANCE** Isoprene is a highly abundant climate-active gas and a carbon source for some bacteria. Analyses of the genes encoding isoprene monooxygenase (IsoMO) indicate this enzyme is a soluble diiron center monooxygenase in the same family of oxygenases as soluble methane monooxygenase, alkene monooxygenase, and toluene monooxygenase. We report the initial biochemical characterization of IsoMO from *Rhodococcus*, the first from any bacterium, describing the challenging purification and reconstitution of *in vitro* activity of its four components. This study lays the foundation for future detailed mechanistic studies of IsoMO, a key enzyme in the global isoprene cycle.

## INTRODUCTION

Isoprene (2-methyl-1,3-butadiene) is a highly volatile hydrocarbon emitted to the atmosphere in a comparable quantity to methane, with over 500 Tg being emitted to the atmosphere every year (1). Emitted primarily by plants, isoprene transformations in the atmosphere have severe consequences, impacting air quality (2), retention of greenhouse gases such as methane (3), and the formation of cloud condensation nuclei (4). Industrial isoprene production has been a focus of research, due to the uses for isoprene in the manufacture of rubber, perfumes, and adhesives (5). The major sink of isoprene is photochemical degradation in the atmosphere, but early studies suggested that soils could also be a significant sink (6, 7). The mechanisms of isoprene degradation by bacteria are still largely uncharacterized, with research thus far focusing on identification of bacteria thought to play an important role in reducing the amount of isoprene entering the atmosphere using DNA-stable isotope probing (8–11) and functional gene probing (12). A range of bacteria have been isolated which are capable of growth on isoprene as a sole carbon and energy source. These include *Variovorax* species isolated from willow soil (9, 13) and oil palm leaves (14), and *Rhodococcus* species from freshwater sediment (15), leaves and soil from poplar (8), and oil palm (9). Less commonly, bacteria of the genera *Gordonia*, Mycobacterium, *Nocardioides*, *Ramlibacter*, and *Sphingopyxis* have also been isolated (9, 10). However, biochemical characterization of the monooxygenase responsible for isoprene degradation has yet to be accomplished.

The most extensively studied isoprene degrading bacterium is the Gram-positive actinobacterium *Rhodococcus* sp. strain AD45 (16). In this bacterium, the initial step in the isoprene degradation pathway is the epoxidation of isoprene to 3,4-epoxy-3-methyl-1-butene by isoprene monooxygenase (IsoMO). This is followed by detoxification of this epoxide through conjugation to glutathione by the glutathione *S*-transferase IsoI, forming 1-hydroxy-2-glutathionyl-2-methyl-3-butene (HGMB). This HGMB is then oxidized by IsoH, an NAD^+^-dependent member of the short-chain dehydrogenase/reductase protein family, to form 2-glutathionyl-2-methyl-3-butenoic acid (GMBA) (15–17). Subsequent steps in the isoprene degradation pathway are uncertain.

**FIG 1:**
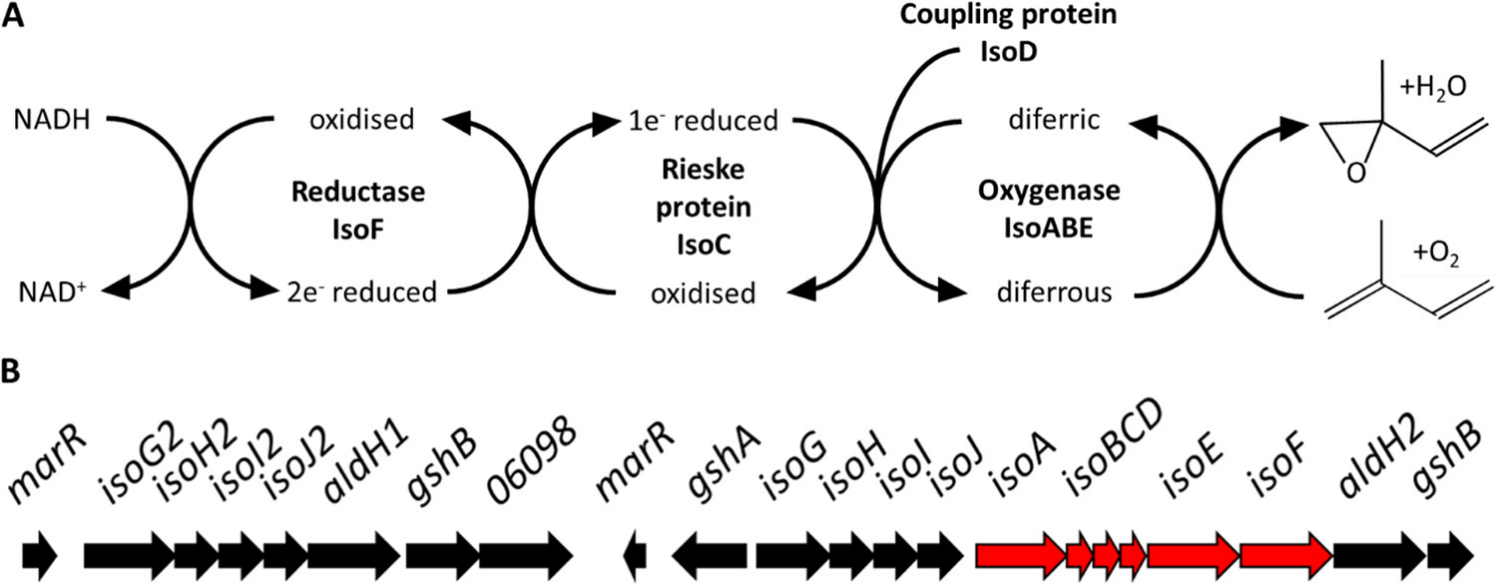
(A and B) Schematic representation of the proposed mechanism of isoprene oxidation by isoprene monooxygenase (A) and the cluster of genes involved in isoprene degradation in *Rhodococcus* sp. AD45, where the genes encoding isoprene monooxygenase are highlighted in red (B).

The gene cluster which encodes all of these proteins is found on a megaplasmid in *Rhodococcus* sp. AD45 (18) (Fig. 1). Genes *isoABCDEF* encode the IsoMO, a soluble diiron monooxygenase (SDIMO) (18), related to methane, toluene, and alkene monooxygenases (19). These SDIMO enzymes are well known for having a wide substrate range, and have been studied for their biotechnological potential and their environmental significance (20). IsoMO of *Rhodococcus* sp. AD45 catalyzes the oxidation of isoprene to 3,4-epoxy-3-methyl-1-butene (15), in contrast to isoprene-degrading *Nocardia* isolates that produce the di-epoxide 1,2-3,4-di-epoxy-butane from isoprene (21). The genes encoding the IsoMO of *Rhodococcus* sp. AD45 indicate a four component SDIMO, similar to the alkene monooxygenase of Xanthobacter autotrophicus Py2 (22) and the toluene-4-monooxygenase of Pseudomonas mendocina KR1 (23). A number of gene clusters encoding IsoMO have been identified in more recently isolated isoprene degraders (8–10, 14) and metagenomic data sets (8, 9, 14). The IsoMO enzymes from Gram-positive bacteria, including *Rhodococcus*, *Gordonia*, and *Nocardioides*, share a high degree of similarity at the sequence level. IsoMO-encoding genes from Gram-negative bacteria, including *Variovorax* and *Sphingopyxis*, group closely together, but cluster separately from those from Gram-positive isoprene degraders, and are more closely related to the alkene monooxygenase from the Gram-negative bacterium Xanthobacter autotrophicus Py2 (13).

The four component IsoMO is likely to comprise an oxygenase (IsoABE), reductase (IsoF), coupling protein (IsoD) and Rieske-type ferredoxin (IsoC) (Fig. 1). Structures derived by X-ray crystallography reveal that the oxygenase components of two other SDIMOs, soluble methane monooxygenase from Methylococcus capsulatus (Bath) (24) and toluene 4-monooxygenase from Pseudomonas mendocina KR1 (25), form dimers in an (αβγ)_2_ arrangement. The alpha subunit of the oxygenase component of SDIMOs (equivalent to IsoA in IsoMO) contains an active site diiron center required for catalytic activity of the enzyme. The beta subunit (IsoE) and the gamma subunit (IsoB) combine with the alpha subunit to form the oxygenase complex.

The reductase component contains two cofactors, an FAD and a [2Fe-2S] cluster, whereas the ferredoxin only contains a Rieske-type [2Fe-2S] cluster. These redox active components are involved in the transfer of electrons, from NADH via the reductase, and the Rieske protein in four-component SDIMOs, to the diiron active site of the oxygenase (23). The reductases of SDIMOs have been repeatedly demonstrated to cause problems in purification and characterization studies. When expressed heterologously in Escherichia coli, the reductase components from toluene/*o*-xylene monooxygenase from Pseudomonas stutzeri OX1 (26) and alkene monooxygenase from Xanthobacter autotrophicus Py2 (27) were either inactive or formed inclusion bodies. In addition, numerous studies have reported a rapid loss of activity in successfully purified reductase, identified in the toluene 4-monooxygenase from Pseudomonas mendocina KR1 (28), and the alkene monooxygenases from Xanthobacter autotrophicus Py2 (22) and Rhodococcus rhodochrous B-276 (29).

The coupling proteins of previously characterized soluble diiron monooxygenases contain no cofactors. They are important for modulating the substrate oxidation rate and regioselectivity, partly by altering the redox potential of the diiron site. This is believed to be achieved through a conformational change in the oxygenase induced by binding of the coupling protein, opening a channel for substrate access to the active site (30). Notably, the coupling protein of the soluble methane monooxygenase from Methylococcus capsulatus (Bath) was consistently purified as a mixture containing an active full-length form and an inactive form due to an N-terminal truncation. While this truncation could be prevented by site directed mutagenesis of the target residues (31), the inactivation of coupling protein, and associated reduction in monooxygenase activity should be considered in the purification of other SDIMOs, including IsoMO.

Previous research has established the importance of the megaplasmid-encoded *isoABCDEF* gene cluster in *Rhodococcus* sp. AD45 for isoprene degradation, with an *isoA* mutant unable to degrade isoprene (18). In addition, the megaplasmid containing genes encoding other enzymes involved in the degradation pathway of isoprene in *Rhodococcus* sp. AD45 has been cured, enabling homologous expression of proteins of interest within the *iso* gene cluster (8). This system was used successfully to express a putative isoprene monooxygenase gene cluster retrieved from a metagenome data set and demonstrate plasmid-encoded isoprene oxidation in whole cells (8), and to express the IsoMO from *Variovorax* sp. WS11 (13). In this study, we aimed to use this homologous expression system, in combination with heterologous expression, to demonstrate the first purification and subsequent initial biochemical characterization of an isoprene monooxygenase, in this case from *Rhodococcus* sp. AD45. We purified and characterized three components of IsoMO, demonstrated the reduction of the Rieske component of IsoMO with the reductase from the related toluene 4-monooxgyenase system, and demonstrated reconstituted activity of IsoMO when purified proteins were added to *Rhodococcus* sp. AD45 cell extract. We have demonstrated formation of the isoprene epoxide from cell lysate of *Rhodococcus* sp. AD45-ID expressing the isoprene monooxygenase gene cluster, and confirmed the activity of the purified oxygenase component when added to alkyne-inhibited *Rhodococcus* sp. AD45 cell extract.

## RESULTS AND DISCUSSION

### Purification and characterization of the oxygenase component (IsoABE) of isoprene monooxygenase from *Rhodococcus* sp. AD45.

**FIG 2:**
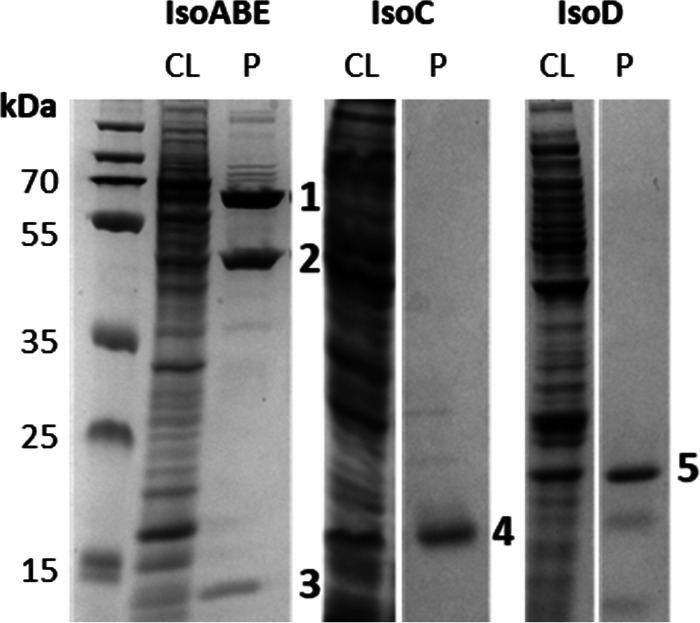
Composite image of SDS-PAGE analysis of IsoABE, IsoC, and IsoD purification. Lanes labeled CL (cell lysate) contain 10 to 15 μg of protein, and lanes labeled P (purified protein) contain 1 to 5 μg of protein. Numbers: 1, IsoA; 2, IsoE; 3, IsoB; 4, IsoC; 5, IsoD.

The oxygenase component of IsoMO from *Rhodococcus* sp. AD45 was expressed in *Rhodococcus* sp. AD45-ID from plasmid pTipQC2S2:isoEx, which encodes the complete IsoMO gene cluster, including an N-terminal StrepII tag on IsoA to aid in purification by affinity chromatography. Production of active isoprene monooxygenase was confirmed by observation of isoprene degradation by cell lysate in the presence of 5 mM NADH. Production of the isoprene epoxide was confirmed by gas chromatography-mass spectrometry (GC-MS) analysis of the cell lysate reaction mixture (see Fig. S1 in the supplemental material). The oxygenase component, comprising IsoA, IsoB, and IsoE, was purified from the cell extract by affinity chromatography and gel filtration chromatography (Fig. 2), yielding 1.4 mg of purified protein per L of culture. The initial elution peak from the gel filtration step was found not to contain the gamma subunit, so fractions from the second elution peak were pooled for subsequent experiments (see Fig. S2). This resulting purified protein complex was examined for the presence of absorbing cofactors and common metal cofactors using UV-visible absorption spectroscopy and inductively coupled plasma mass spectrometry (ICP-MS), respectively. Absorption spectroscopy of IsoABE revealed a lack of spectral features above 300 nm (see Fig. S3), consistent with previous SDIMO oxygenase preparations, including those from the methane monooxygenase of Methylococcus capsulatus (Bath) (32), toluene 2-monooxygenase from Burkholderia cepacia G4 (33), and toluene/*o*-xylene monooxygenase from Pseudomonas stutzeri OX1 (26).

ICP-MS analysis of purified IsoABE was used to confirm the presence of the predicted diiron center, see Table 1. Initial ICP-MS analysis of IsoABE eluted from the StrepTactin column and exchanged into the buffer used for gel filtration using Corning Spin-X UF concentrators indicated the presence of 4.81 Fe per monomer, which is higher than the predicted 2 Fe/monomer (results not shown). The sample was therefore subjected to a further gel filtration step, to ensure the removal of adventitiously bound Fe. Data from this preparation indicated the presence of 1.12 ± 0.02 Fe per monomer of protein, which is lower than the predicted amount. For comparison, the oxygenase component of the toluene 2-monooxygenase from Burkholderia cepacia G4 was found to contain 2.95 equivalents of Fe per monomer of oxygenase (33), and the toluene/*o*-xylene monooxygenase from Pseudomonas stutzeri OX1 was found to contain 1.7 equivalents of iron per monomer of oxygenase (26). Thus, purified IsoMO oxygenase is deficient in Fe cofactor and would be expected to exhibit decreased activity as a result.

**TABLE 1:** Results of ICP-MS analysis of purified oxygenase (IsoABE) and Rieske protein (IsoC) components from isoprene monooxygenase of *Rhodococcus* sp. AD45

Component	No. of atoms per protein molecule ± SD	
	IsoABE	IsoC
S^*a*^	33	6
Fe	1.12 ± 0.02	1.80 ± 0.06
Mg	0.01 ± 0.04	0.04 ± 0.01
Mn	0.00 ± 0.00	0.00 ± 0.00
Co	0.00 ± 0.00	0.00 ± 0.00
Ni	0.01 ± 0.00	0.02 ± 0.00
Cu	0.11 ± 0.00	0.15 ± 0.04
Zn	0.04 ± 0.00	0.00 ± 0.02

a Sulfur concentrations were used to determine protein concentrations (and thus equivalents of metals present), based on the predicted amino acid sequence, combined with LC-MS confirmation in the case of IsoC. The cysteine/methionine contents of IsoABE and IsoC are 7/26 and 3/3, respectively.

### Purification and characterization of the Rieske protein (IsoC) component of isoprene monooxygenase from *Rhodococcus* sp. AD45.

**FIG 3:**
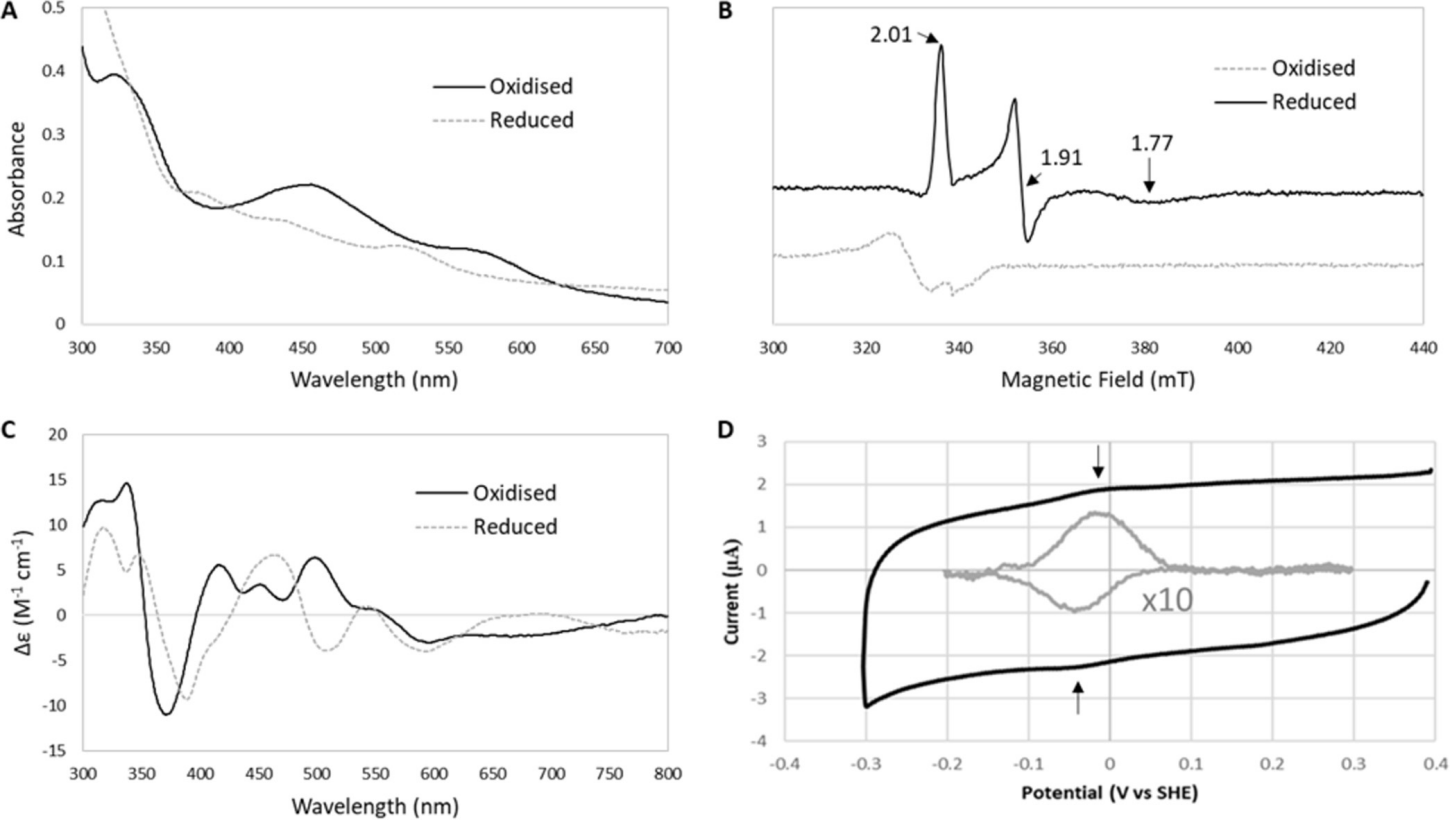
Characterization of the Rieske protein component (IsoC) of isoprene monooxygenase from *Rhodococcus* sp. AD45. (A) Absorption spectra of oxidized (solid line) and reduced (dotted line) IsoC. (B) EPR spectra of oxidized (as isolated) and reduced IsoC. (C) Circular dichroism spectra of oxidized (solid line) and reduced (dotted line) IsoC. (D) Cyclic voltammograms of IsoC produced from the average of three concurrent scans (black line), including a baseline corrected and multiplied (×10) cyclic voltammogram of IsoC showing the reductive (−40 mV) and oxidative (−12 mV) peaks (gray line). Scans were recorded at 20 mV s^−1^ in 50 mM HEPES–50 mM NaCl (pH 7) buffer at 20°C.

The Rieske protein component (IsoC) of IsoMO from *Rhodococcus* sp. AD45 was expressed in *Rhodococcus* sp. AD45-ID using plasmid pTipQC1:*isoC* (see Materials and Methods), which allowed the production of an N-terminally His_6_-tagged IsoC. This was not attempted in Escherichia coli, as we believed the homologous expression system was most likely to yield protein with correctly incorporated Rieske-type [2Fe-2S] cluster. This protein was purified from the cell lysate under aerobic conditions by affinity chromatography (see Fig. S4) and gel filtration chromatography (see Fig. S5; yield, 0.4 mg per L of culture), after which densitometric analysis of the SDS-PAGE gel indicated that the protein was >90% pure. The resulting IsoC sample was analyzed to investigate the presence of cofactors by UV-visible absorption, circular dichroism, and electron paramagnetic resonance (EPR) spectroscopies. Absorption spectroscopy of IsoC as isolated contained clear maxima at 323 and 454 nm, with a shoulder at 575 nm, consistent with IsoC containing a [2Fe-2S] cluster in its oxidized, 2+ state. Upon reduction with sodium dithionite, the spectrum changed significantly, with maxima at 382, 431, and 515 nm (Fig. 3A), indicative of reduction of the cluster to its reduced, 1+ state. The features of the UV-visible absorption spectrum of oxidized and reduced IsoC are consistent with the presence of a Rieske-type [2Fe-2S] cluster, as has been identified in other four-component SDIMO systems, including the alkene monooxygenase of Xanthobacter autotrophicus Py2 (22), the toluene 4-monooxygenase of Pseudomonas mendocina KR1 (23), and toluene/*o*-xylene monooxygenase from Pseudomonas stutzeri OX1 (26).

The circular dichroism spectrum of oxidized IsoC (as isolated) had maxima at 315, 337, 416, 451, and 498 nm and minima at 370, 436, 471, and 595 nm. In contrast, the reduced IsoC spectrum featured maxima at 318, 347, 464, 542, and 680 nm and minima at 336, 389, 509, and 594 nm (Fig. 3C). The circular dichroism spectral characteristics are also consistent with a [2Fe-2S] Rieske-type cluster, similar to those reported for the alkene monooxygenase from Xanthobacter autotrophicus Py2 (22), phthalate oxygenase from Pseudomonas cepacia (34), and sulredoxin from the archaeon *Sulfolobus* sp. strain 7 (35). One study detailing the similarities between the Rieske protein components of benzene dioxygenase from Pseudomonas putida ML2 and the *bc*_1_ complex from bovine heart mitochondria identified a characteristic strong negative band at ∼500 nm upon reduction of the proteins (36), which was clearly observed in the circular dichroism spectrum of reduced IsoC.

The EPR spectrum of as isolated, oxidized IsoC contained only a signal (*g* value at 2.06) characteristic of Cu^2+^ (Fig. 3B), which was likely to be adventitiously bound, consistent with a small amount of Cu detected by ICP-MS (Table 1). In contrast, the reduced spectrum contained signals with *g* values at 2.01, 1.91, and 1.77 (Fig. 3B). These are characteristic of a Rieske-type [2Fe-2S] cluster in its reduced (+1) state, and are similar to those obtained for the Rieske protein of other four-component SDIMOs, including toluene 4-monooxygenase from Pseudomonas mendocina KR1 (23) and alkene monooxygenase from Xanthobacter autotrophicus Py2 (22).

ICP-MS analysis of purified IsoC confirmed the presence of the [2Fe-2S] cluster (Table 1), and indicated that >90% of the IsoC protein contained a cluster, based on the 1.85 Fe per IsoC and 2 sulfur per protein in addition to that due to the cysteine and methionine content of the protein. Using the protein concentration determined using the ICP-MS data, extinction coefficients for IsoC were calculated to be ɛ_280 nm_ = 29,060 ± 2,070 M^−1 ^cm^−1^, and ɛ_460 nm_ = 6,990 ± 550 M^−1 ^cm^−1^; these values are consistent with those of the Rieske protein component of alkene monooxygenase from Xanthobacter autotrophicus Py2, reported as 26,683 M^−1 ^cm^−1^ and 7,230 M^−1 ^cm^−1^ at 280 and 460 nm, respectively (22).

Protein film electrochemistry was used to determine the midpoint reduction potential of IsoC. Protein films were prepared on the pyrolytic graphite edge electrodes coated with poly-lysine. Clear oxidative and reductive peaks were observed at −12 mV and −41 mV (respectively) when the protein was present but not in its absence. The peak shape was consistent with a one electron redox process with a midpoint potential of −26 ± 10 mV versus standard hydrogen electrode (SHE) at pH 7.0 (Fig. 3D). This value is higher than observed for other Rieske proteins from four-component soluble diiron monooxygenases. The closest literature value is for the Rieske protein of alkene monooxygenase from Xanthobacter autotrophicus Py2 at −49 ± 10 mV versus SHE at pH 7.0 (22), perhaps unsurprising since this is the most closely related system to IsoMO, based on homology of the amino acid sequences (16). However, other SDIMO Rieske proteins possess lower midpoint potentials, including toluene/*o*-xylene monooxygenase (−130 mV versus SHE at pH 7.0) (37), toluene 4-monooxygenase (−173 mV versus SHE at pH 7.0) (38). Despite this, the value reported here for IsoC is within the range previously reported for Rieske proteins more generally, lying between −155 and +312 mV versus SHE which were reported for the Rieske component of benzene dioxygenase of Pseudomonas cepacia and the *bc*_1_ complex of bovine heart mitochondria, respectively (36).

### Purification and characterization of the coupling protein (IsoD) of isoprene monooxygenase from *Rhodococcus* sp. AD45.

The coupling protein component (IsoD) of IsoMO from *Rhodococcus* sp. AD45 was expressed in Escherichia coli Rosetta2/(pLysS) using plasmid pET51b:*isoD*, which allowed the production of an N-terminally StrepII-tagged IsoD (see Materials and Methods). Purification of IsoD from the cell lysate was attempted using affinity, anion exchange, hydrophobic interaction and gel filtration chromatography, yielding 3.6 mg protein per L of culture (see Fig. S6). The use of anion exchange and hydrophobic interaction chromatography was not found to significantly increase the purity of the sample, and densitometric analysis of an SDS-PAGE gel indicated that the preparation was approximately 75% pure after affinity and gel filtration chromatography steps (Fig. 2). As with IsoABE, the absorption spectrum demonstrated no features above 300 nm, suggesting the absence of any colored cofactors (see Fig. S7). The lack of such cofactors in soluble diiron monooxygenase coupling proteins is well documented, for example those from Xanthobacter autotrophicus Py2 (22) and Methylosinus trichosporium OB3b (39).

Liquid chromatography-mass spectrometry (LC-MS) analysis of the purified coupling protein demonstrated a clear peak at 14,603 Da, as expected assuming that the N-terminal methionine had been removed. While small amounts of lower-molecular-mass polypeptides were present, no peaks were at a high enough level to suggest the truncation of IsoD during the purification process. The lack of a truncation site here was of particular interest, as previously it was shown that truncation of the N terminus of the coupling protein of soluble methane monooxygenase inactivated the enzyme complex, and the importance of a specific cleavage site between Met and Gly at the N terminus was demonstrated by site directed mutagenesis (31). The N terminus of coupling proteins from soluble diiron monooxygenases have consistently been reported to contain unstructured tails of various lengths (40–46); however, the use of N-terminal tags and protein fusions to aid in protein purification has not prevented reconstitutions of the active complex in a number of soluble diiron monooxygenase systems (27, 40, 47, 48).

### Attempted purification of the reductase (IsoF) of isoprene monooxygenase from *Rhodococcus* sp. AD45, and reduction of IsoC with the reductase of toluene 4-monooxygenase from *Pseudomonas mendocina* KR1.

The reductase components of soluble diiron monooxygenases have been reported to be difficult to purify, containing a [2Fe-2S] cluster and FAD cofactor, and often either failing to purify in active form, or with activity being rapidly lost (22, 23, 26–29, 49). Numerous attempts were made to purify the reductase component (IsoF) of IsoMO from *Rhodococcus* sp. AD45. These included both homologous expression in *Rhodococcus* sp. AD45-ID and heterologous expression in Escherichia coli Rosetta2/(pLysS). Expression of His-tagged, untagged, glutathione *S*-transferase (GST)-fusion and maltose binding protein (MBP)-fusion IsoF resulted in protein which was either expressed entirely in inclusion bodies, expressed so poorly it was impossible to purify, or when purified did not contain the predicted cofactors or demonstrate the ability to reduce IsoC.

**FIG 4:**
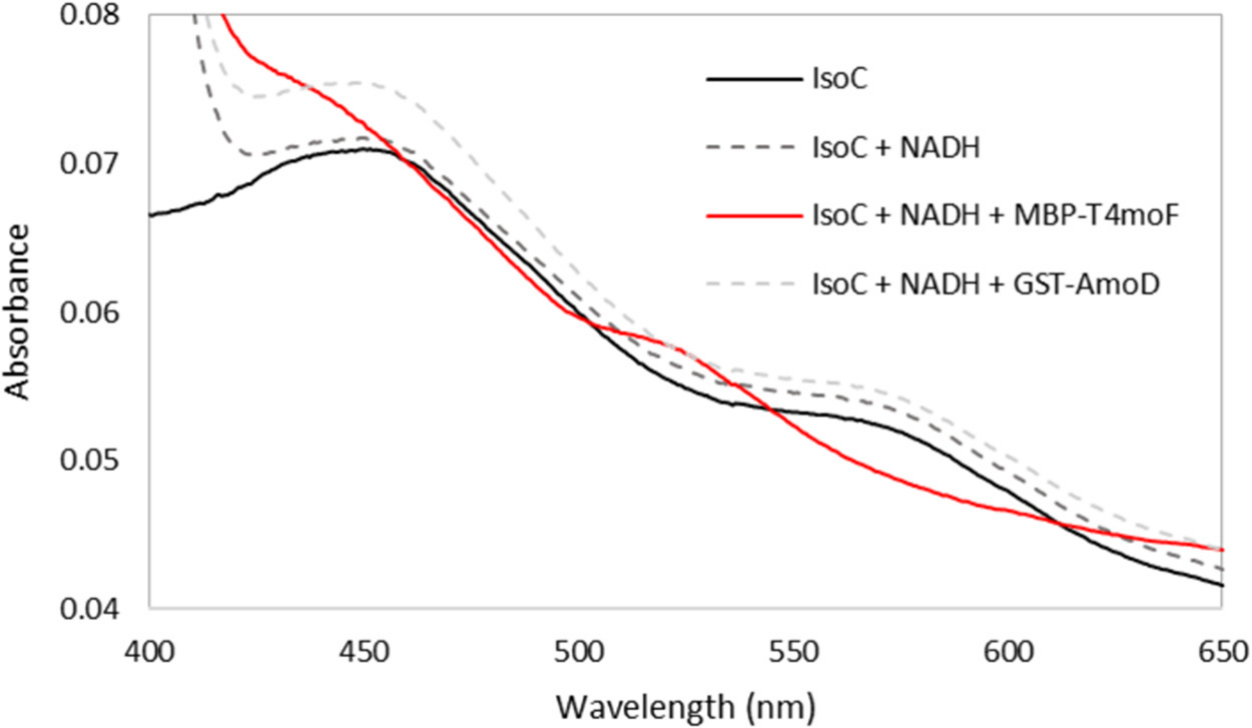
Absorption spectra of the Rieske protein (IsoC) in the presence of NADH and reductases from toluene 4-monooxygenase (as the MBP fusion protein [MBP-T4moF]) and alkene monooxygenase (as the GST fusion protein [GST-AmoD]).

Due to these drawbacks, plasmids for the expression of reductase components from the related SDIMOs, toluene 4 monooxygenase (T4MO) of Pseudomonas mendocina KR1 (50) and alkene monooxygenase (AMO) from Rhodococcus rhodochrous B-276 (48) were acquired as generous gifts from Brian Fox (University of Wisconsin—Madison) and Tom Smith (Sheffield Hallam University), respectively. The T4MO reductase (T4MOF) was expressed with an N-terminal MBP fusion in E. coli Rosetta2/(pLysS), and purified using anion exchange (Q-Sepharose) and affinity (MBPTrap) chromatography, as described previously (50) (see Fig. S8). The AMO reductase (AMOD) was also expressed in E. coli Rosetta2/(pLysS), but with an N-terminal GST fusion. This was purified using a GSTrap affinity chromatography column, as previously published (48) (see Fig. S8). These purified reductase fusion proteins were examined, using absorption spectroscopy, for their ability to catalyze the reduction of IsoC by NADH. Only the MBP-T4MOF fusion protein was found to be capable of this reaction (Fig. 4).

The difficulty in producing functional reductase proteins has been documented previously in the case of alkene monooxygenase from Xanthobacter autotrophicus Py2, where a recombinant T4MO was also utilized in reconstitution experiments to demonstrate activity of the purified alkene monooxygenase components (27). Use of alternative reductases has also been reported in reconstitution experiments with other SDIMOs (51, 52); however, this has mostly been to investigate the effect that alternate SDIMO components have on stereoselectivity, rather than being driven by necessity. The fact that the recombinant reductase from T4MO, but not the recombinant reductase from AMO, could reduce IsoC is interesting. The T4MO system is a four-component soluble diiron monooxygenase system, like IsoMO, whereas the AMO system from Rhodococcus rhodochrous B-276 is a three-component system, which means that no Rieske protein is required. Despite this, the two alternate reductases (T4MOF and AMOD) share a similar level of sequence identity (30 and 33%, respectively) with IsoF. Further elucidation of the residues which determine the interaction of the T4MOF with the Rieske protein (IsoC) of IsoMO presents an intriguing avenue for further study, to investigate whether switching of various reductase components between soluble diiron monooxygenase systems alters stereoselectivity of the monooxygenase reactions.

### Reconstitution of the active isoprene monooxygenase complex from *Rhodococcus* sp. AD45 in the presence of *Rhodococcus* sp. AD45 cell lysate.

IsoMO activity was monitored by measuring the consumption of isoprene in the headspace of 25-mL vials using a fast isoprene sensor, since the epoxide produced is unstable in aqueous solutions (15). A 1.5-mL suspension containing 5 or 10 μM concentrations (each) of IsoABE, IsoC, and IsoD and 1 μM MBP-T4MOF in 150 mM phosphate buffer containing 300 mM NaCl and 5 mM dithiothreitol (DTT; pH 7.0) was prepared. Approximately 200 parts per million by volume (ppmv) isoprene was added to the headspace, and 5 mM NADH was added to initiate the reaction. However, no isoprene oxidation activity was observed under these conditions.

**FIG 5:**
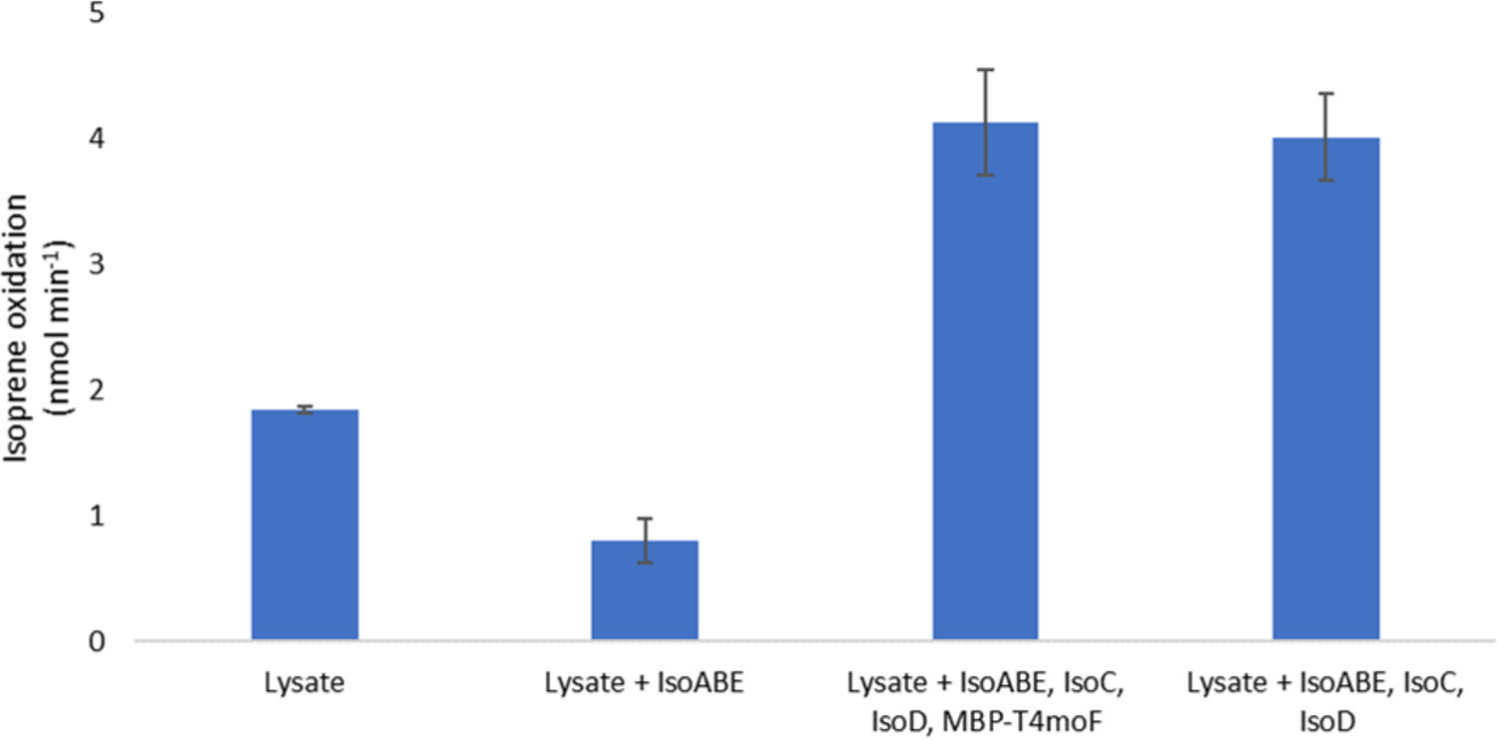
Rate of isoprene oxidation measured using assays containing cell lysate of *Rhodococcus* sp. AD45 grown on isoprene as a carbon source, with the addition of various purified components from isoprene monooxygenase (IsoABE, IsoC, and IsoD) or an MBP fusion of the reductase from toluene 4-monooxygenase of Pseudomonas mendocina KR1 (MBP-T4MOF). All assays contained 5 mM DTT and 5 mM NADH.

To check the activity of IsoMO after cell lysis, the clarified cell extract from a cell suspension of *Rhodococcus* sp. AD45 grown on isoprene was tested. In all subsequent assays, 1 mL of this cell extract, containing 4.5 to 5.5 mg of protein, was included. This assay mixture was made to a 1.5-mL total volume by the addition of assay buffer (as described above). This resulted in isoprene epoxidation at a rate of 1.84 ± 0.02 nmol min^−1^. The addition of purified oxygenase component at a concentration of 5 μM reduced this rate to 0.80 ± 0.18 nmol min^−1^. However, the addition of IsoABE, IsoC, and IsoD at 5 μM and of MBP-T4MOF at 1 μM increased the activity to 4.13 ± 0.42 nmol min^−1^. This activity was not reduced by the omission of MBP-T4MOF, found to be 4.01 ± 0.35 nmol min^−1^ (Fig. 5). An abiotic control confirmed the observed isoprene depletion was due to enzymatic activity, with isoprene depletion measured at 0.01 ± 0.05 nmol min^−1^. These data suggest that although the MBP-T4MOF could reduce IsoC, the endogenous IsoF component may still be required to restore activity. The inability of the MBP-T4MOF to confer monooxygenase activity despite clearly demonstrating the ability to reduce IsoC could be due to a lack of dissociation from the Rieske protein component. This could prevent IsoC from interacting with IsoABE, thus preventing the subsequent electron transfer step required for activity.

Subtracting the rate of isoprene depletion by the cell lysate from that of the reconstituted complex resulted in a specific activity of isoprene oxidation determined to be 4.10 nmol min^−1 ^mg IsoABE^−1^ for the purified oxygenase component. Previously, the activity of reconstituted alkene monooxygenase from Xanthobacter autotrophicus Py2 on propylene was recorded as 6.25 nmol min^−1^ (53), which, based on the published assay concentrations used, would suggest a specific activity of 19.5 nmol min^−1 ^mg oxygenase^−1^ (53). Furthermore, the soluble methane monooxygenase from Methylococcus capsulatus (Bath) had a specific activity of 82.3 nmol min^−1 ^mg oxygenase^−1^ using ethylene as a substrate (54). Thus, the observed activity for IsoMO was low in comparison to published data for other SDIMOs, which may be due to the low Fe cofactor incorporation reported for the oxygenase component in the ICP-MS data above. The use of cell lysate to compensate for the absence of purified reductase also likely affected the observed rate.

**FIG 6:**
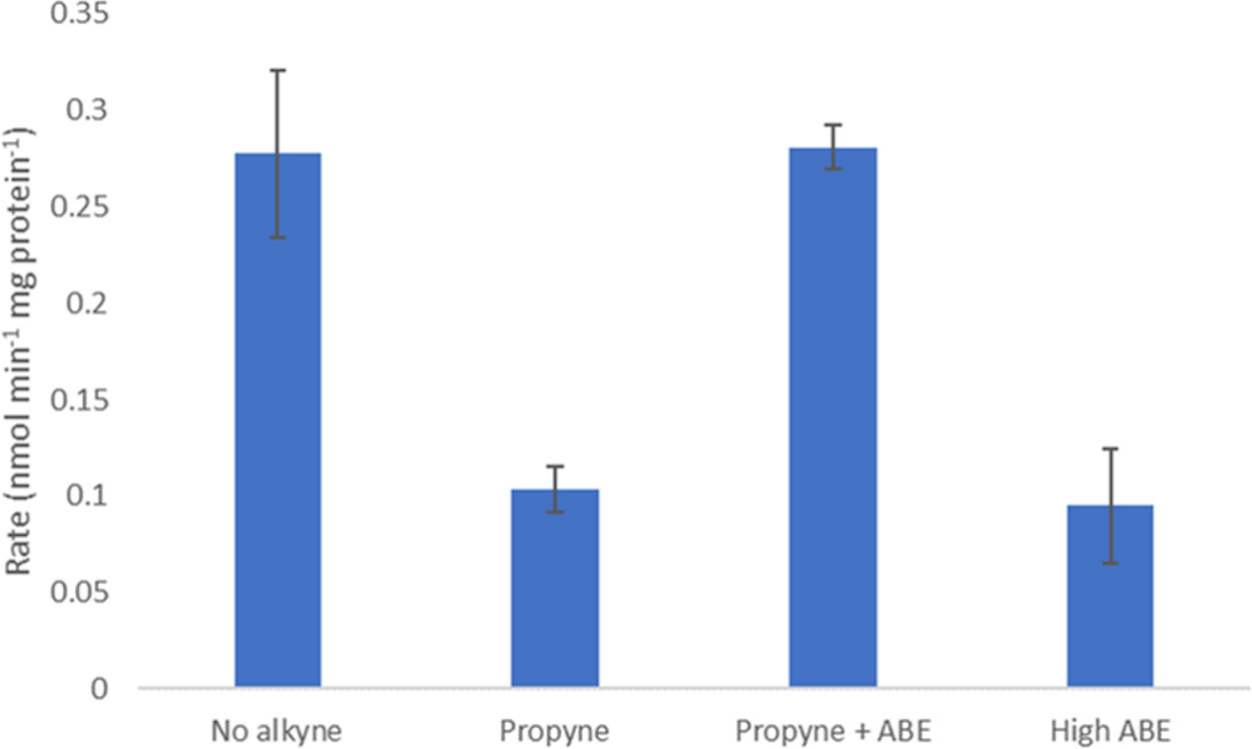
Rate of isoprene oxidation by cell lysate of *Rhodococcus* sp. AD45 grown on isoprene as a carbon source. All assays were preincubated for 15 min in the presence (propyne, propyne + ABE, and high ABE) or absence (no alkyne) of 1% (vol/vol) headspace propyne, 5 mM NADH, and 5 mM DTT. This was followed by headspace exchange and the addition of IsoABE (0.5 mg in propyne + ABE, 1.25 mg in high ABE).

The reduction in rate observed upon addition of an excess of purified oxygenase was unexpected. To confirm the activity of the purified oxygenase and effect of high levels, the aforementioned experiment was repeated with propyne-inhibited *Rhodococcus* sp. AD45 cell lysate, as used to demonstrate the activity of the purified oxygenase component of alkene monooxygenase from Xanthobacter autotrophicus Py2 (22). The cell lysate was inhibited by preincubation with 1% (vol/vol) propyne for 15 min, followed by removal of the alkyne by exchanging the headspace of the vial. A control was performed by running this preincubation and headspace exchange without the alkyne present to prove that the inhibitory effect was not due to these additional steps. The addition of 0.5 mg of purified IsoABE was found to return the activity to the previously observed level; however, 1.25 mg of IsoABE did not (Fig. 6). This could be due to the absence of additional coupling protein, which is known to be important in substrate access to the active site (30). The specific activity of isoprene oxidation when 0.5 mg of purified protein was added was determined to be 3.17 nmol min^−1 ^mg IsoABE^−1^, which is consistent with the assay using the noninhibited cell lysate.

### Conclusions.

In summary, we have demonstrated that the purified oxygenase component of IsoMO, IsoABE, from *Rhodococcus* sp. AD45 is active and contains the predicted diiron nonheme cofactor. The Rieske protein component, IsoC, when expressed using a homologous system and purified with an N-terminal His tag, was capable of redox cycling, contained the predicted [2Fe-2S] Rieske-type cluster and possessed a midpoint potential of −26 ± 10 mV versus SHE, which was within the normal range for other Rieske proteins (−155 to +312 mV versus SHE). The coupling protein component IsoD could be expressed heterologously, though difficulties in obtaining pure protein were encountered. The coupling protein was not inactivated by the type of truncation observed in the soluble methane monooxygenase system. While the purification of the reductase component IsoF was even more troublesome, the T4MO reductase (containing a MBP-fusion) was found to be capable of reducing the Rieske protein component. However, this may not fully compensate for the endogenous reductase, since the omission of this component did not lower the observed IsoMO activity when the other three components were added to *Rhodococcus* sp. AD45-ID cell lysate. This system for purifying and monitoring the activity of IsoMO will allow for further studies on the enzymology of isoprene oxidation.

## MATERIALS AND METHODS

### Cloning.

The PCR primers in Table 2 were used to amplify the corresponding genes from *Rhodococcus* sp. AD45 genomic DNA using Q5 High Fidelity polymerase (New England Biosciences), according to manufacturer’s instructions. The resulting PCR products were purified using a Roche HighPure PCR product purification kit and ligated to the pJET1.2/blunt vector using the CloneJET kit (Thermo Fisher), according to the manufacturer’s instructions. The resulting mixture was used to transform E. coli TOP10 chemically competent cells (Invitrogen) according to the manufacturer’s instructions and then plated on LB medium containing ampicillin (100 μg mL^−1^). A geneJET plasmid miniprep kit (Thermo Scientific) was used to extract plasmid from transformants, and the gene of interest was cut from this using the appropriate restriction enzymes (Table 2). The target vector for each gene was digested using the appropriate enzymes, and both digested insert and vector were cut from agarose gels and purified using a HighPure PCR product purification kit (Roche). The insert and vector were ligated using T4 DNA ligase (Thermo Fisher), and the resulting ligation mix was used to transform E. coli TOP10 cells and then plated on LB medium containing ampicillin (as above). Plasmid minipreps were prepared as outlined above, and the predicted sequences confirmed by sequencing (Eurofins, Germany).

**TABLE 2:** Primers used in this study

Target gene	Target vector	Primer	Sequence (5′–3′)	Restriction enzyme
*isoC*	pTipQC1	isoCF	ATATATCATATGGCTGACGGTCAGGACA	NdeI
		isoCR	ATATATGGATCCTACTTCAGTGTTGCGCCT	BamHI
*isoD*	pET51b	51isoDF	TATATAGGATCCGATGGCCATTGTCGATATGGATGC	BamHI
		51isoDR	TATATAAAGCTTTCATGACTTCTCCTGTCCAGGGT	HindIII
	pTipQC2	QCS2F	TCCCATGGCCTTCTCGAATTGTGGATGAGACCAGCCCATATGTATATCTCCTTCTTAAAG	NcoI
		QCS2R	GCTTCTCGGGTGTACATATC	BsrGI
*isoABCDEF*	pTipQC2S2	isoExF	ATATACATGTTGTTGAACAGGGACGATTGGTACG	PciI
		isoExR	ATATGGATCCGTTAAGCGATGAAGCGGTCGAAG	BamHI

For expression of the oxygenase component, plasmid pTipQC2 was first modified to enable expression of protein incorporating an N-terminal StrepII tag. The 198-bp BsrGI*/*NcoI fragment was excised by restriction digestion and replaced by ligation into the same sites of a similar sequence incorporating a StrepII-encoding sequence, generated by PCR from pTipQC2 using pTipQCS2F and pTipQCS2R primers (Table 2), resulting in plasmid pTipQC2S2. The entire monooxygenase gene sequence *isoABCDEF* was PCR amplified from *Rhodococcus* sp. AD45 using the primers isoExF/isoExR, excised from pJet1.2 using PciI and BamHI, and then cloned into the NcoI and BamHI sites of pTipQC2S2, resulting in pTipQC2S2:isoEx (Table 3).

**TABLE 3:** Strains and plasmids used in this study

Strain or plasmid	Description	Source or reference
Strains		
E. coli TOP10	Strain used for cloning and plasmid maintenance	Invitrogen
E. coli Rosetta 2/(pLysS)	Strain used for protein expression	Sigma-Aldrich
*Rhodococcus* sp. AD45	Wild-type strain	15
*Rhodococcus* sp. AD45-ID	Strain AD45 cured of megaplasmid	8
		
Plasmids		
pGEX-AmoD	Expression vector for AmoD from *Rhodococcus rhodochrous* B-276	48
pVP68KT4moF	Expression vector for T4moF from *Pseudomonas mendocina* KR1	50
pJETblunt	Amp^r^ cloning vector	Thermo Fisher Scientific
pTipQC1	Chl^r^ inducible expression vector, functional in *Rhodococcus*, MCS type 1	57
pTipQC2	Chl^r^ inducible expression vector, functional in *Rhodococcus*, MCS type 2	57
pTipQC2S2	pTipQC2 with N-terminal StrepII tag sequence	This study
pTipQC2S2:isoEx	pTipQC2S2 containing *isoABCDEF* from strain AD45	This study
pTipQC1:*isoC*	pTipQC1 containing *isoC* from strain AD45	This study
pET51b	Amp^r^ expression vector	Novagen
pET51b:*isoD*	pET51b containing *isoD* from strain AD45	This study

### Expression of recombinant proteins and preparation of cell lysate.

Chemically competent E. coli Rosetta2/(pLysS) (Sigma-Aldrich) cells were transformed according to manufacturer’s instructions and then plated on LB medium containing ampicillin (as described above) and chloramphenicol (35 μg mL^−1^). Electrocompetent *Rhodococcus* sp. AD45-ID cells were prepared and transformed as previously described (8). E. coli Rosetta2/(pLysS) containing the pET51b:*isoD*, pGEX-AmoD, or pVP68KT4moF plasmid were grown at 37°C to an optical density at 540 nm (OD_540_) of 0.6 to 0.8, followed by induction with 50 μM IPTG (isopropyl-β-d-thiogalactopyranoside). Cells containing pET51b:*isoD* were grown at 37°C for a further 3 h before harvesting by centrifugation (8,000 × *g*, 4°C, 20 min). Cells containing pGEX-AmoD or pVP68KT4moF were grown at 25°C for a further 18 h before harvest by centrifugation (8,000 × *g*, 4°C, 20 min). *Rhodococcus* sp. AD45-ID cells containing pTipQC2S2:isoEx or pTipQC1:*isoC* were grown at 30°C to an OD_540_ of ∼0.6, followed by induction with 0.6 μM thiostrepton, from a 12 μM stock dissolved in dimethyl sulfoxide. The cells were then grown at 25°C for a further 18 h before harvest by centrifugation (8,000 × *g*, 4°C, 20 min). Cell pellets were snap-frozen in liquid nitrogen and stored at −80°C until use. E. coli cells were broken by sonication (15 s on and 45 s off for a total of 10 min), whereas *Rhodococcus* sp. AD45 cells were broken by three passes through a French Pressure cell at 20,000 lb/in^2^ (137 MPa).

### Purification of the oxygenase and coupling protein components of isoprene monooxygenase from *Rhodococcus* sp. AD45.

*Rhodococcus* sp. AD45-ID cell pellets which had expressed *isoABCDEF* (pTipQC2S2:isoEx) or coupling protein alone (pET51b:*isoD*) were thawed and resuspended in a binding buffer containing 100 mM Tris-HCl and 150 mM NaCl (and 1 mM EDTA in buffers for IsoD purification) at pH 8.0. The cell extract was prepared and applied to a 5 mL of Strep-Tactin XT column using an AKTApure HPLC system with UNICORN7.0 software. Elution of proteins was carried out in a single step using elution buffer containing the same components as the binding buffer, with the addition of 50 mM biotin. The resulting protein fractions were analyzed by SDS-PAGE, and those containing the polypeptide band of interest were pooled and concentrated by centrifugation using 20 mL of 5-kDa-molecular-weight cutoff Spin-X UF columns (Corning) to a volume of <2.5 mL. The concentrated protein was applied to a Superdex HiLoad 26/600 200 pg size exclusion column (Cytiva) equilibrated in 50 mM phosphate buffer containing 150 mM NaCl (pH 7.0). Fraction samples were analyzed by SDS-PAGE, and those containing the polypeptide band(s) of interest were pooled, concentrated as described above, snap-frozen in liquid nitrogen, and stored at −80°C until needed.

### Purification of the Rieske protein component of isoprene monooxygenase from *Rhodococcus* sp. AD45.

Cell pellets containing the Rieske protein component expressed from pTipQC1:*isoC* were thawed and resuspended in buffer containing 50 mM Tris-HCl, 300 mM NaCl, and 30 mM imidazole (pH 7.4). Cell extract was applied to a 5-mL HisTrap column, and elution of bound protein was achieved using a 50-mL gradient from 30 to 500 mM imidazole. Fractions that were brown in color, indicating the presence of the [2Fe-2S] cluster, were pooled and concentrated as outlined above. This protein was then subjected to size exclusion chromatography, as outlined above for the IsoMO oxygenase.

### Purification of alternative reductase components.

Cell pellets containing the reductases of toluene 4 monooxygenase (MBP-T4MOF) from Pseudomonas mendocina KR1 (50) and alkene monooxygenase (GST-AMOD) from Rhodococcus rhodochrous B-276 (48) were purified as described previously.

### Spectroscopic characterization of purified proteins.

EPR measurements were made using an X-band Bruker EMX EPR spectrometer equipped with a helium flow cryostat (Oxford Instruments). EPR spectra were measured at 10 K at the following instrumental settings: microwave frequency, 9.471 GHz; microwave power, 3.18 mW; modulation frequency, 100 kHz; modulation amplitude, 5 G; time constant, 82 ms; scan rate, 22.6 G s^−1^; single scan per spectrum. Protein samples for EPR spectroscopy were prepared in an anaerobic glove box. The protein was reduced by the addition of an excess of sodium dithionite, and samples were flash-frozen in liquid nitrogen.

UV-visible spectra were recorded and colorimetric assays performed using a Shimadzu UV-1800 spectrophotometer, quartz cuvettes, and UVprobe software (Shimadzu). A JASCO model J810 circular dichrograph was used to measure CD spectra for the wavelength range 250 to 800 nm. Protein samples were prepared as for EPR spectroscopy but transferred to anaerobic quartz cuvettes for measurement at ambient temperature.

### Analysis of purified IsoMO components by mass spectrometry.

LC-MS was conducted using a Bruker microQTof-QIII electrospray ionization time-of-flight mass spectrometer. The instrument was calibrated in the *m/z* range 300 to 2,000 using ESI-L low concentration tuning mix (Agilent Technologies). Samples were prepared by 10-fold dilution of 50 μM protein solution with 0.1% (vol/vol) formic acid and 2% (vol/vol) acetonitrile to 500 μL. An UltiMate 3000 HPLC system (Dionex) with autosampler was used to apply 20-μL protein samples to a ProSwift reversed-phase RP-1S column (Dionex) at 25°C. The elution was performed at 200 mL min^−1^ over a gradient using solvents: line A contained 0.1% formic acid, and line B contained acetonitrile and 0.1% formic acid. An isocratic wash (2% B, 0 to 2 min) was performed, followed by a linear gradient from 2 to 100% B (2 to 12 min), an isocratic wash (100% B, 12 to 14 min), and column re-equilibration (2% B, 14 to 15 min). Mass spectra were acquired throughout using the following parameters: dry gas flow, 8 L min^−1^; nebulizer gas pressure, 0.8 bar; dry gas, 240°C; capillary voltage, 4,500 V; offset, 500 V; and collision RF, 650 Vpp. Mass spectra from elution volumes manually selected for their proximity to the predicted protein mass were averaged and deconvoluted using a deconvolution algorithm in Compass DataAnalysis version 4.1 (Bruker Daltonik).

Samples for ICP-MS analysis were prepared by pipetting purified protein samples (1 to 20 μM, 500 μL) into clean microcentrifuge tubes. To the protein samples, 200 μL of ultrapure nitric acid was added, followed by 200 μL of ultrapure hydrogen peroxide. These were then sealed and heated at 30 to 40°C overnight. These samples were then centrifuged (13,000 × *g*, 10 min) to remove any undissolved protein sample. After this, 500 μL of digested sample was transferred to a 10-mL Falcon tube, and both 500 μL of ^103^Rh internal standard and 4 mL ultrapure Milli-Q water were added. These samples were then applied to the Thermo Elemental X Series 1 instrument (Thermo Scientific) using an associated Cetac ASX 520 autosampler. Accuracy was verified by the ^103^Rh measurement, and data were only collected where this was recorded at 80 to 100% of the expected amount. PlasmaLab software was used to analyze the data.

### Determination of the midpoint reduction potential of the Rieske protein (IsoC) component using protein film electrochemistry.

Protein film electrochemistry of the Rieske protein (IsoC) was carried out in a three-electrode cell configuration using a stationary pyrolytic graphite edge working electrode, an Ag/AgCl (saturated KCl) reference electrode, and a Pt wire counter electrode (55). The working electrode was prepared by three cycles of polishing with alumina slurry followed by sonicating and washing in ultrapure milli-Q water. A 4-μL aliquot of 1 mg mL^−1^ poly-l-lysine solution was applied to the electrode and left to adsorb for 5 min before excess was removed. A solution of IsoC in 50 mM HEPES–50 mM NaCl (pH 7) buffer was placed on to the surface of the electrode and left to adsorb for 5 min before the excess was removed. The electrode was transferred to an N_2_-filled anaerobic glove box and placed into an electrochemical cell containing 4 mL of buffer (50 mM HEPES-50 mM NaCl [pH 7.0]). Cyclic voltammetry was performed using a PGSTAT12 (Metrohm Autolab) potentiostat under the control of NOVA 1.4 software. Cyclic voltammograms were recorded between −0.3 and 0.4 V versus SHE at a scan rate of 20 mV s^−1^, at 20°C. The resulting data were analyzed using Nova 1.4 software, and the oxidative and reductive peaks were calculated after the subtraction of an appropriate baseline. The midpoint potential was then calculated as the average of the reductive and oxidative peaks. Potentials are reported versus SHE by the addition of 0.197 V to the values measured using Ag/AgCl electrode.

### Isoprene oxidation assays.

Isoprene oxidation was monitored by measuring the concentration of isoprene in the headspace using a Fast Isoprene Sensor (Hills Scientific) (56). Assays were performed using 25-mL vials containing 1 mL of *Rhodococcus* sp. AD45 cell extract (4.5 to 5.5 mg mL^−1^ protein) and 0.5 mL of 50 mM phosphate buffer (pH 7.0) and/or purified protein components at a final concentration of 5 μM, in the presence of 5 mM DTT. Cell extract was prepared by the resuspension of frozen cell pellets in 50 mM phosphate buffer (pH 7.0) and 5 mM DTT. The cells were broken by three passes through a French pressure cell at 20,000 lb/in^2^ (137 MPa). Unbroken cells and cell debris were removed by centrifugation at 16,000 × *g* (4°C, 30 min). Protein concentration was determined using a Bradford-based assay (Bio-Rad). Once sealed with butyl stoppers, ∼200 ppmv isoprene was added to the headspace, vials were transferred to a 30°C water bath with orbital shaking and left to equilibrate for 3 min. The reaction was initiated by the addition of 5 mM NADH, and 50-μL headspace samples were analyzed every 3 min for a total of 15 min using a Fast Isoprene Sensor (Hills Scientific). The data were analyzed using the QtiPlot program (version 5.6.1, Ion Vasilief), and compared to standards ranging between 70 and 500 ppmv isoprene to determine the rate of isoprene oxidation.

### Detection of epoxyisoprene by GC-MS.

For detection of epoxy isoprene, samples (0.5 mL) were extracted with an equal volume of diethyl ether and analyzed by gas chromatography – mass spectrometry, using a GC-MS-QP2010s instrument (Shimadzu, Milton Keynes, UK) equipped with an AOC-20s autosampler. Helium was used as carrier gas with 0.8 mL min^−1^ flow through the column (SHIM-5MS, 30 m × 0.25 mm, 0.25-μm film thickness; Shimadzu). The inlet was held at 250°C, and the oven was maintained at 30°C for 3 min and then ramped to 150°C at 25°C min^−1^ and held for 1 min. The interface and ion source were at 250 and 200°C, respectively. Sample injection volume was 1 μL, in split mode (1:10 split). Initially, mass spectra were obtained in scan mode, detecting ions in the range *m/z* 35 to 500 and compared to the National Institute of Standards and Technology Mass Spectral Database (NIST 08). The retention time of epoxy isoprene was 3.24 min. For quantification, selected ion monitoring mode was used, targeting ions 39, 43, and 53 (*m/z*). Quantification was performed using GC-MS solution v.2.50 (Shimadzu) against a calibration curve consisting of four 10-fold dilutions of epoxy isoprene obtained from Merck (Gillingham, UK) (0.857 to 857 μg mL^−1^).

### Data availability.

The gene sequences used to design primers to clone *isoABCDEF* from *Rhodococcus* sp. strain AD45 for this study are freely available from the National Centre for Biotechnology Information, accession number CM003191 (18).
