# Supplementary material for: Purification and Characterization of the Isoprene Monooxygenase from Rhodococcus sp. Strain AD45
Source: Appl Environ Microbiol. 2022 Mar 14;88(7):e00029-22. doi: 10.1128/aem.00029-22 (PMC9004368; doi:10.1128/aem.00029-22)
Supplement: Supplemental file 1 — Fig. S1 to S8. Download aem.00029-22-s0001.pdf, PDF file, 0.3 MB [file aem.00029-22-s0001.pdf]

SUPPLEMENTARY

Contents:

Figure S1: GC-MS Spectra of epoxy isoprene from cell lysate (above) and NIST library (below).

Figure S*2*: Purification of IsoABE using affinity and size exclusion chromatography.

Figure S3: Absorption spectrum of the oxygenase component (IsoABE) of isoprene monooxygenase from *Rhodococcus* sp. AD45

Figure S4: Polypeptide profile of affinity chromatography purification of IsoC, the Rieske protein component of isoprene monooxygenase from *Rhodococcus* sp. AD45.

Figure S5: Polypeptide profile of gel filtration chromatography purification of IsoC, the Rieske protein component of isoprene monooxygenase from *Rhodococcus* sp. AD45.

Figure S6: Polypeptide profiles of A) affinity chromatography purification of IsoD, the coupling protein component of isoprene monooxygenase from *Rhodococcus* sp. AD45.

Figure S7: Absorption spectrum of the coupling protein (IsoD) of isoprene monooxygenase from *Rhodococcus* sp. AD45

Figure S8: Purification of MBP-T4moF and GST-AmoD fusion proteins

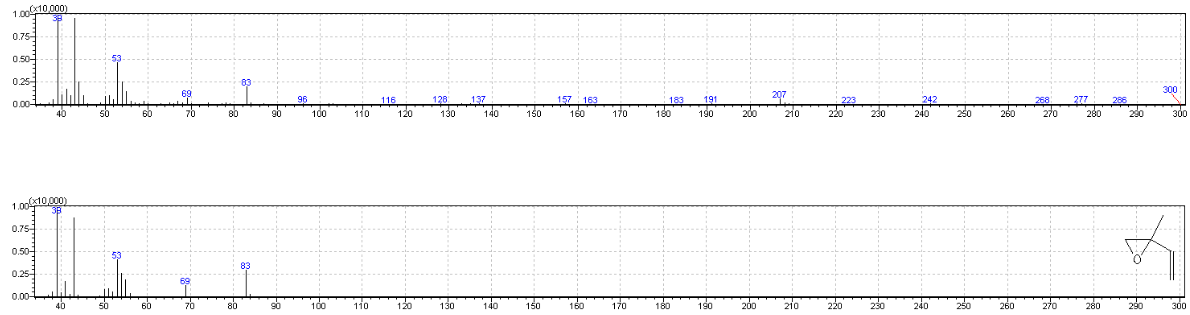

Figure S1: GC-MS Spectra of epoxy isoprene from cell lysate (above) and NIST library (below).

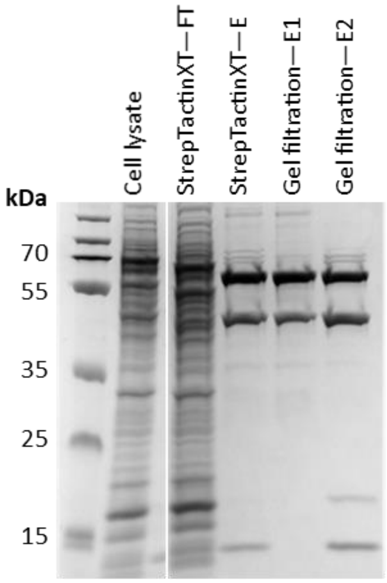

Figure S2: Purification of IsoABE using affinity and size exclusion chromatography. Polypeptide profiles of fractions were visualised using SDS-PAGE and InstantBlue staining. Cell extract and StrepTactinXT FT (flow through) samples contained (10 µg protein). Pooled StrepTactinXT and gel filtration E (elution) samples contained 5 µg protein.

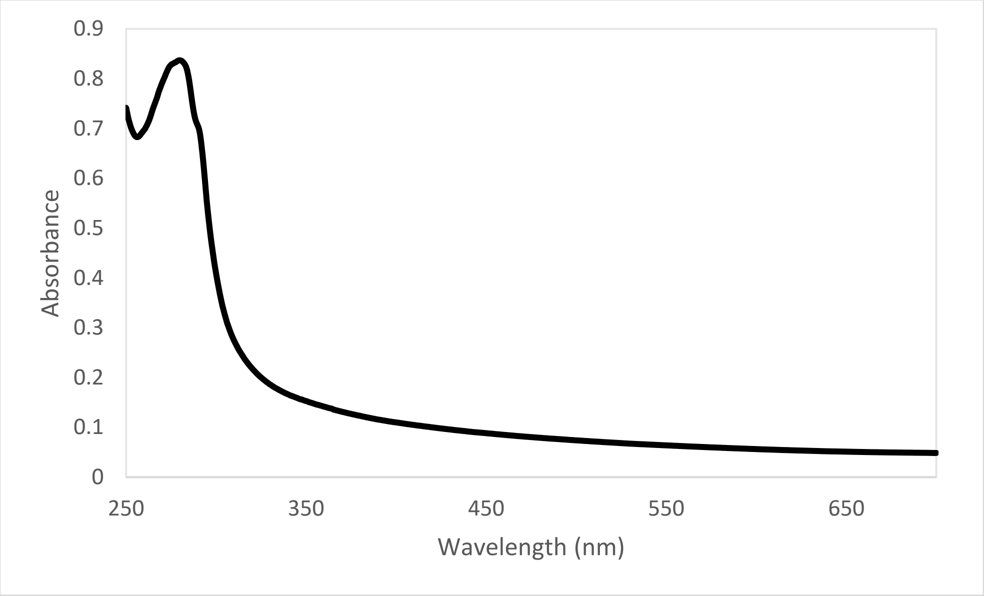

Figure S3: Absorption spectrum of the oxygenase component (IsoABE) of isoprene monooxygenase from *Rhodococcus* sp. AD45, as purified from *Rhodococcus* sp. AD45-ID containing the pTipQC2S2:IsoEx expression plasmid. The oxygenase component was purified by affinity chromatography and gel filtration chromatography.

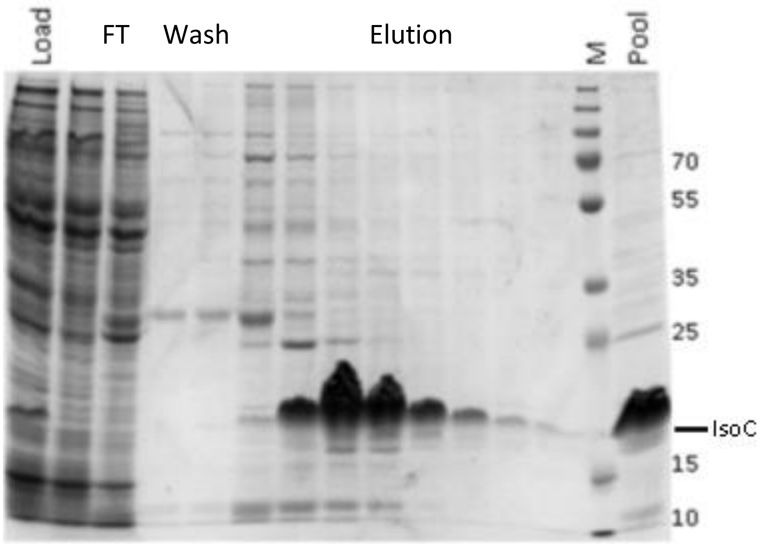

Figure S4: Polypeptide profile of affinity chromatography purification of IsoC, the Rieske protein component of isoprene monooxygenase from *Rhodococcus* sp. AD45. Load is the cell lysate (15 µg protein), FT is the column flow through (15 µg protein). Elution was performed over a gradient from 40 – 500 mM imidazole (10 µL samples). Samples brown in colour, indicative of the presence of the [2Fe-2S] cluster, were combined and are labelled here as "pool" (5 µg protein).

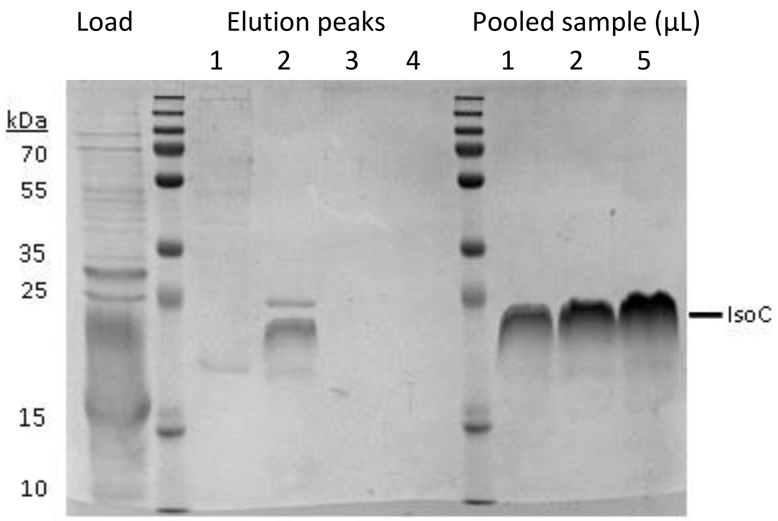

Figure S5: Polypeptide profile of gel filtration chromatography purification of IsoC, the Rieske protein component of isoprene monooxygenase from *Rhodococcus* sp. AD45. Load is the pooled elution fractions from affinity chromatography (10 µg protein), elution peaks are the samples from each absorption peak measured by the AKTA HPLC Unicorn software (20 µL samples). The pooled gel filtration-purified samples were analysed at varying concentrations on the right hand side (protein concentration 2.5 µg µL^{-1}, numbers on gel correspond to µL of sample loaded).

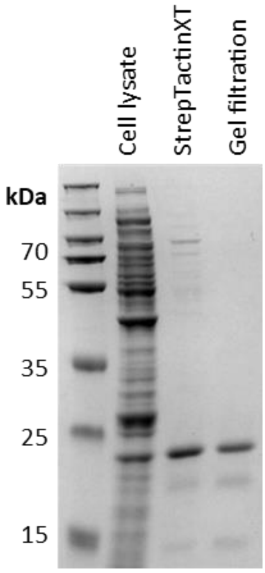

Figure S6: Polypeptide profiles of purification of IsoD, the coupling protein component of isoprene monooxygenase from *Rhodococcus* sp. AD45. StrepTactin and Gel filtration are the pooled elution fractions from the corresponding column. Cell lysate sample contains 10 µg protein, and the purified protein samples contain 2 µg protein.

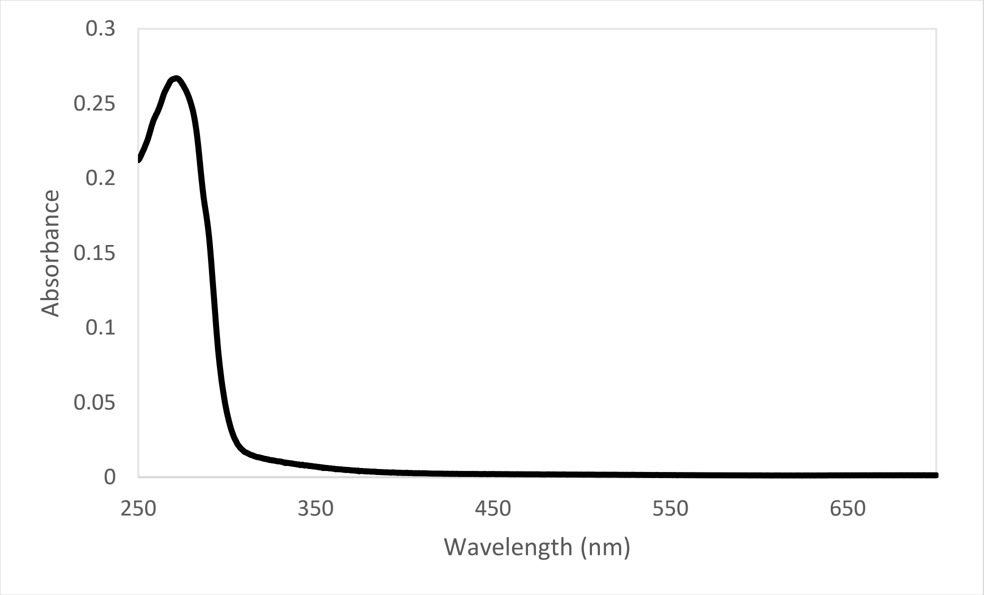

Figure S7: Absorption spectrum of the coupling protein (IsoD) of isoprene monooxygenase from *Rhodococcus* sp. AD45, as purified from *Escherichia coli* Rosetta2 (pLysS) containing the pET51b:isoD expression plasmid. The coupling protein was purified by affinity chromatography and gel filtration chromatography.

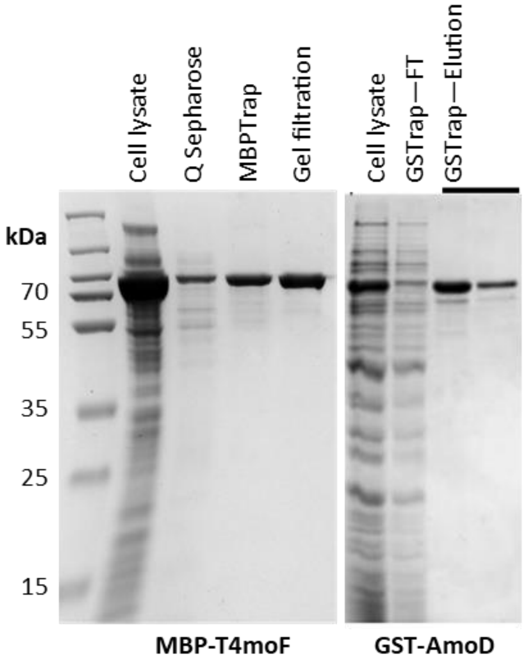

Figure S8: Purification of MBP-T4moF and GST-AmoD fusion proteins. Steps in MBP-T4moF purification are labelled with the column which they were eluted from. GST-AmoD was purified by GSTrap column alone, and column flow through is labelled FT. Cell lysate samples contain 10-15 µg protein, whereas purified protein samples contain 1-2 µg protein.
